# Supplementary material for: Immunodominant proteins P1 and P40/P90 from human pathogen Mycoplasma pneumoniae
Source: Nat Commun. 2020 Oct 14;11:5188. doi: 10.1038/s41467-020-18777-y (PMC7560827; doi:10.1038/s41467-020-18777-y)
Supplement: Supplementary file 1 — Supplementary Information [file 41467_2020_18777_MOESM1_ESM.pdf]

Supplementary Information

Immunodominant proteins P1 and P40/P90 from human pathogen *Mycoplasma pneumoniae*

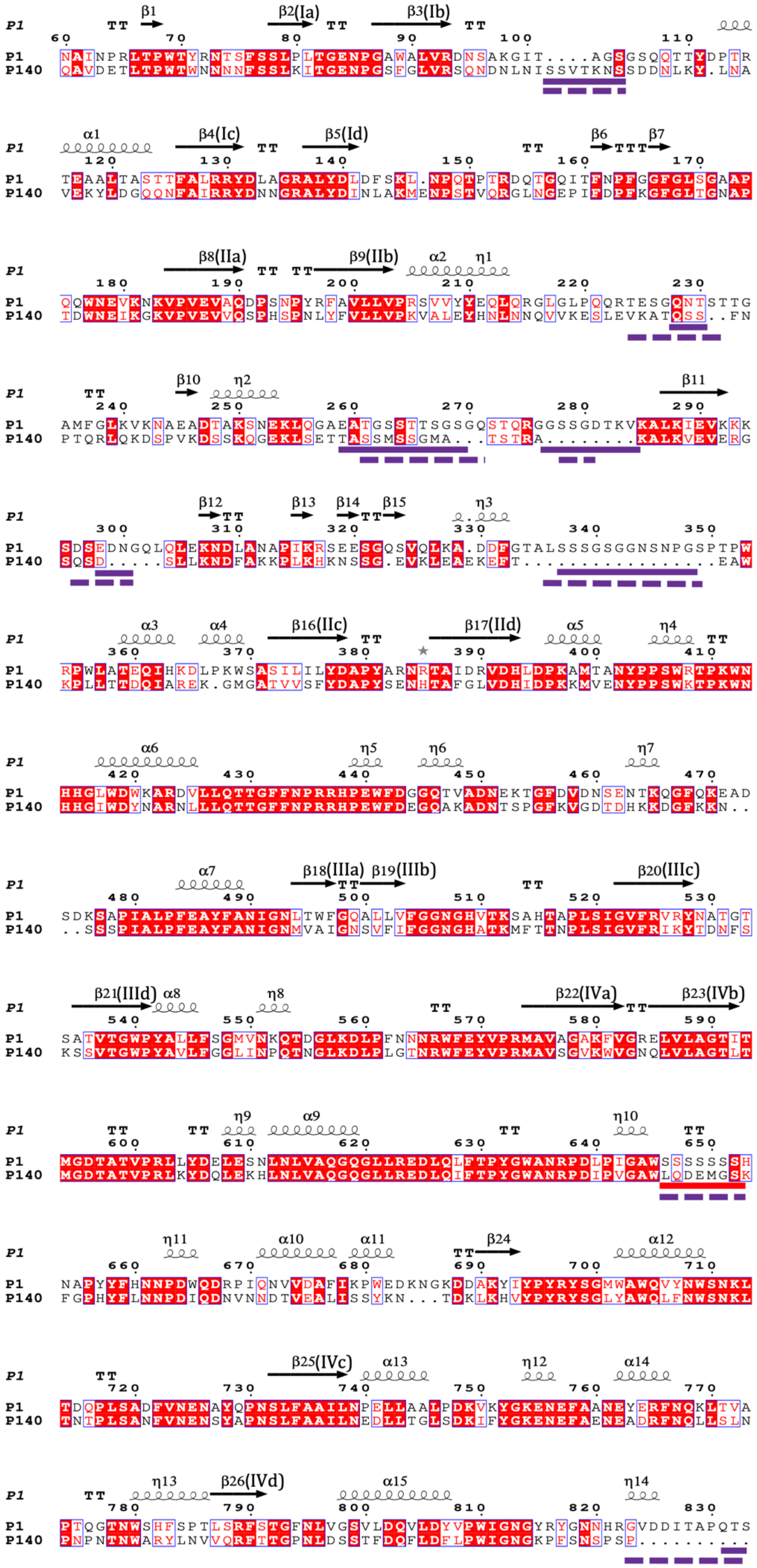

P1 β1 β2(Ia) β3(Ib)
60 70 80 90 100 110
P1 NAINPRLTPWTYRNTSFSSLPLTGENPGAWALVRDNSAKGIT....AGSGSQQTTYDPTR
P140 QAVDETLTPWTWNNNNFSSLKITGENPGSFGLVRSQNDNLNISSVTKNSSDDNLKY.LNA
P1 α1 β4(Ic) β5(Id) β6 β7
120 130 140 150 160 170
P1 TEAALTASTTFALRRYDLAGRALYDLDFSKL.NPQTPTRDQTGQITFNPFGGFGLSGAAP
P140 VEKYLDGQQNFAIRRYDNNGRALYDINLAKMENPSTVQRGLNGEPIFDPFKGFGLTGNAP
P1 β8(IIa) β9(IIb) α2 η1
180 190 200 210 220 230
P1 QQWNEVKNKVPVEVAQDPSNPYRFAVLLVPRSVVYYEQLQRGLGLPQQRTESGQNTSTTG
P140 TDWNEIKGKVPVEVVQSPHSPNLYFVLLVPKVALEYHNLNNQVVKESLEVKATQSS..FN
P1 β10 η2 β11
240 250 260 270 280 290
P1 AMFGLKVKNAEADTAKSNEKLQGAEATGSSTTSGSGQSTQRGGSSGDTKVKALKIEVKKK
P140 PTQRLQKDSPVKDSSKQGEKLSETTASSMSSGMA...TSTRA........KALKVEVERG
P1 β12 β13 β14 β15 η3
300 310 320 330 340 350
P1 SDSEDNGQLQLEKNDLANAPIKRSEESGQSVQLKA.DDFGTALSSSGSGGNSNPGSPTPW
P140 SQSD.....SLLKNDFAKKPLKHKNSSG.EVKLEAEKEFT.................EAW
P1 α3 α4 β16(IIc) β17(IId) α5 η4
360 370 380 390 400 410
P1 RPWLATEQIHKDLPKWSASILILYDAPYARNRTAIDRVDHLDPKAMTANYPPSWRTPKWN
P140 KPLLTTDQIAREK.GMGATVVSFYDAPYSENHTAFGLVDHIDPKKMVENYPPSWKTPKWN
P1 α6 η5 η6 η7
420 430 440 450 460 470
P1 HHGLWDWKARDVLLQTTGFFNPRRHPEWFDGGQTVADNEKTGFDVDNSENTKQGFQKEAD
P140 HHGIWDYNARNLLLQTTGFFNPRRHPEWFDEGQAKADNTSPGFKVGDTDHKKDGFKKN..
P1 α7 β18(IIIa) β19(IIIb) β20(IIIc)
480 490 500 510 520 530
P1 SDKSAPIALPFEAYFANIGNLTWFGQALLVFGGNGHVTKSAHTAPLSIGVFRVRYNATGT
P140 ..SSSPIALPFEAYFANIGNMVAIGNSVFIFGGNGHATKMFTTNPLSIGVFRIKYTDNFS
P1 β21(IIId) α8 η8 β22(IVa) β23(IVb)
540 550 560 570 580 590
P1 SATVTGWPYALLFSGMVNKQTDGLKDLPFNNNRWFEYVPRMAVAGAKFVGRELVLAGTIT
P140 KSSVTGWPYAVLFGGLINPQTNGLKDLPLGTNRWFEYVPRMAVSGVKWVGNQLVLAGTLT
P1 η9 α9 η10
600 610 620 630 640 650
P1 MGDTATVPRLLYDELESNLNLVAQGQGLLREDLQLFTPYGWANRPDLPIGAWSSSSSSSH
P140 MGDTATVPRLKYDQLEKHLNLVAQGQGLLREDLQIFTPYGWANRPDIPVGAWLQDEMGSK
P1 η11 α10 α11 β24 α12
660 670 680 690 700 710
P1 NAPYYFHNNPDWQDRPIQNVVDAFIKPWEDKNGKDDAKYIYPYRYSGMWAWQVYNWSNKL
P140 FGPHYFLNNPDIQDNVNNDTVEALISSYKN...TDKLKHVVPYRYSGLYAWQLFNWSNKL
P1 β25(IVc) α13 η12 α14
720 730 740 750 760 770
P1 TDQPLSADFVNENAYQPNSLFAAILNPELLAALPDKVKYGKENEFAANEYERFNQKLTVA
P140 TNTPLSANFVNENSYAPNSLFAAILNEDLLTGLSDKIFYGKENEFAENEADRFNQLLSLN
P1 η13 β26(IVd) α15 η14
780 790 800 810 820 830
P1 PTQGTNWSHFSPTLSRFSTGFNLVGSVLDQVLDYVPWIGNGYRYGNNHRGVDDITAPQTS
P140 PNPNTNWARYLNVVQRFTTGPNLDSSTFDQFLDFLPWIGNGKPFSNSPSP..........

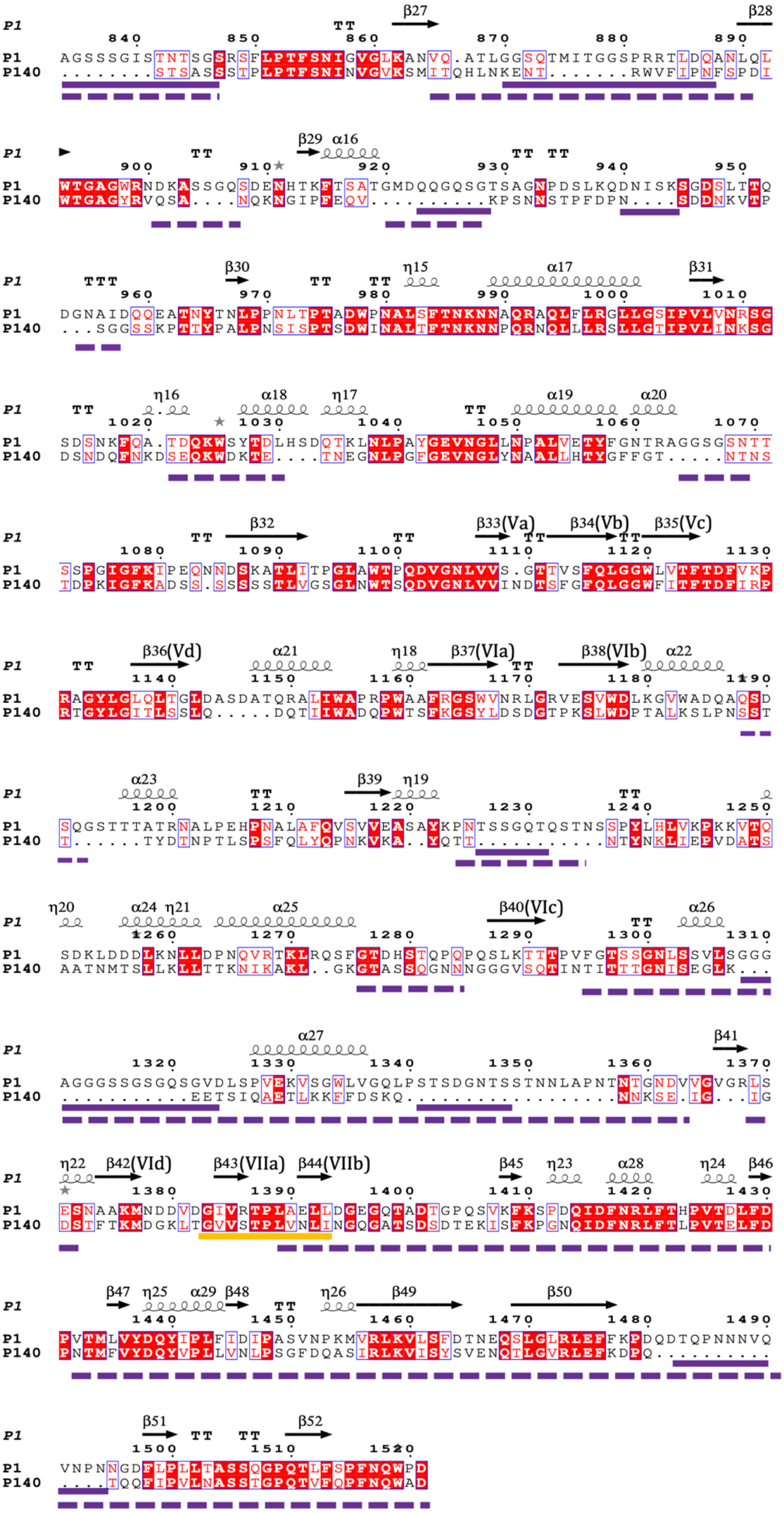
P1 AGSSSGISTNTSGSRSFLPTFSNIGVGLKANVQ.ATLGGSQTMITGGSPRRTLDQANLQL
P140 .........STSASSSTPLPTFSNINVGVKSMITQHLNKENT.......RWVFIPNFSPDI
P1 WTGAGWRNDKASSGQSDENHTKFTSATGMDQQGQSGTSAGNPDSLKQDNISKSGDSLTTQ
P140 WTGAGYRVQSA....NQKNGIPFEQV..........KPSNNSTPFDPN....SDDNKVTP
P1 DGNAIDQQEATNYTNLPPNLTPTADWPNALSFTNKNNAQRAQLFLRGLLGSIPVLVNRSG
P140 ...SGGSSKPTTYPALPNSISPTSDWINALTFTNKNNPQRNQLLLRSLLGTIPVLINKSG
P1 SDSNKFQA.TDQKWSYTDLHSDQTKLNLPAYGEVNGLLNPALVETYFGNTRAGGSGSNTT
P140 DSNDQFNKDSEQKWDKTE....TNEGNLPGFGEVNGLYNAALLHTYGFFGT.....NTNS
P1 SSPGIGFKIPEQNNDSKATLITPGLAWTPQDVGNLVVS.GTTVSFQLGGWLVTFTDFVKP
P140 TDPKIGFKADSS.SSSSSTLVGSGLNWTSQDVGNLVVINDTSFGFQLGGWFITFTDFIRP
P1 RAGYLGLQLTGLDASDATQRALIWAPRPWAAFRGSWVNRLGRVESVWDLKGVWADQAQSD
P140 RTGYLGITLSSLQ.....DQTIIWADQPWTSFKGSYLDSDGTPKSLWDPTALKSLPNSST
P1 SQGSTTTATRNALPEHPNALAFQVSVVEASAYKPNTSSGQTQSTNSSPYLHLVKPKKVTQ
P140 T......TYDTNPTLSPSFQLYQPNKVKA..YQTT...........NTYNKLIEPVDATS
P1 SDKLDDDLKNLLDPNQVRTKLRQSFGTDHSTQPQPQSLKTTTPVFGTSSGNLSSVLSGGG
P140 AATNMTSLLKLLTTKNIKAKL..GKGTASSQGNNGGGVSQTINTITTTGNISEGLK...
P1 AGGGSSGSGQSGVDLSPVEKVSGWLVGQLPSTSTSDGNTSSTNNLAPNTNTGNDVVGVGRLS
P140 ...........EETSIQAETLKKFFDSKQ.....................NNKSE.IG...IG
P1 ESNAAKMNDDVDGIVRTPLAELLDGEGQTADTGPQSVKFKSPDQIDFNRLFTHPVTDLFD
P140 DSTFTKMDGKLTGVVSTPLVNLINGQGATSDSDTEKISFKPGNQIDFNRLFTLPVTELFD
P1 PVTMLVYDQYIPLFIDIPASVNPKMVRLKVLSFDTNEQSLGLRLEFFKPDQDTQPNNNVQ
P140 PNTMFVYDQYVPLLVNLPSGFDQASIRLKVISYSVENQTLGVRLEFKDPQ..........
P1 VNPNNGDFLPLLTASSQGPQTLFSPFNQWPD
P140 ....TQQFIPVLNASSTGPQTVFQPFNQWAD

Supplementary Figure 1. **Structural sequence alignments of P1**

Structural sequence alignments obtained by the superposition between P1 and the orthologous protein P140 from *M. genitalium* and represented with program Espript [1]. The DSSP assignment of secondary structure elements for P1 are represented above the sequence, as squiggles (α-helices), arrows (β-strands) and TT (turns). Missing residues, corresponding to disordered regions, in the X-ray or in the Cryo-EM structures are indicated with a continuous or dashed purple underline, respectively. The AGT repeats are underlined red and the cytoadherence epitope in orange.

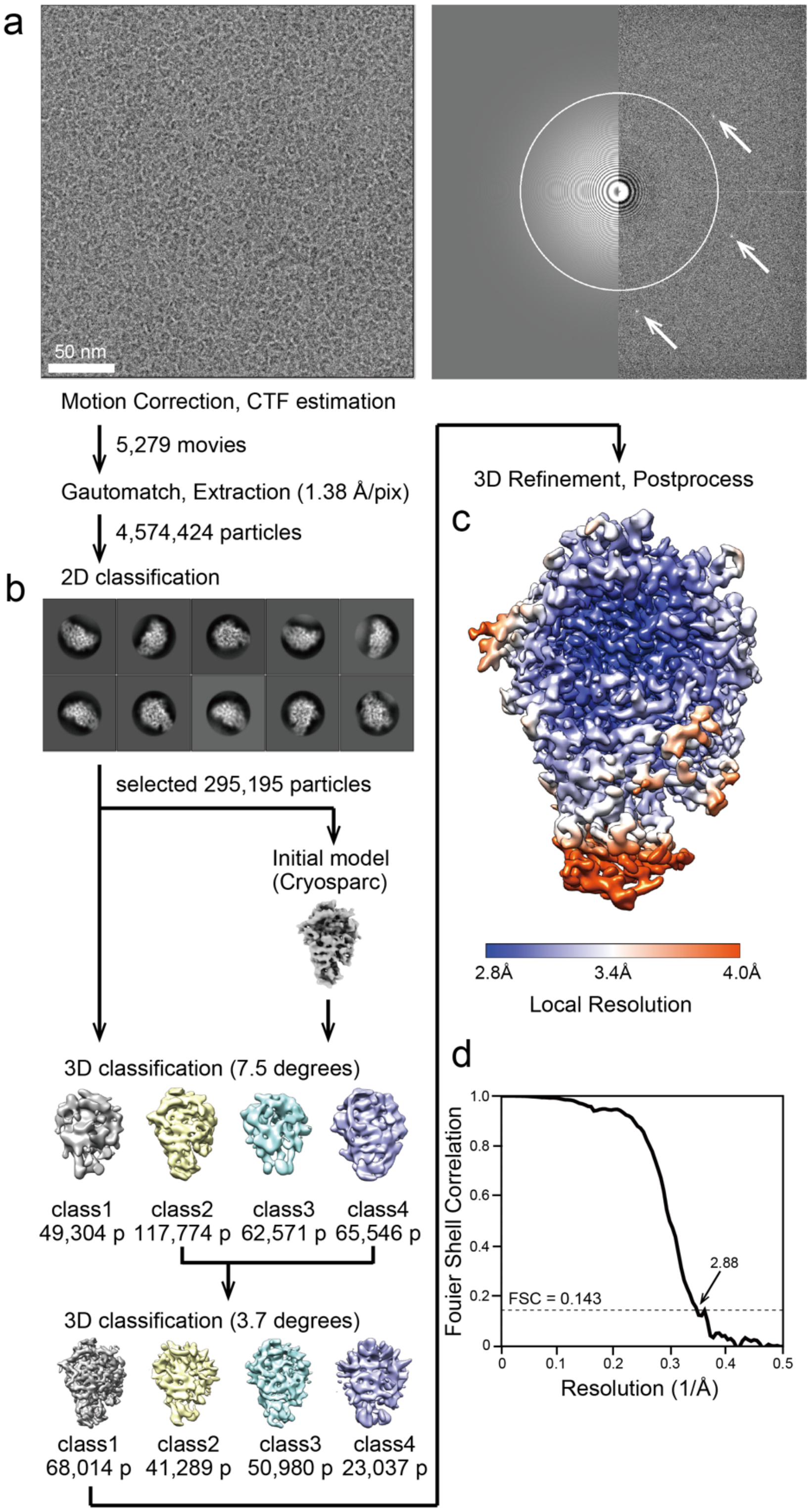
a
50 nm
Motion Correction, CTF estimation
5,279 movies
Gautomatch, Extraction (1.38 Å/pix)
4,574,424 particles
b
2D classification
selected 295,195 particles
Initial model
(Cryosparc)
3D classification (7.5 degrees)
class1
49,304 p
class2
117,774 p
class3
62,571 p
class4
65,546 p
3D classification (3.7 degrees)
class1
68,014 p
class2
41,289 p
class3
50,980 p
class4
23,037 p
3D Refinement, Postprocess
c
2.8Å
3.4Å
4.0Å
Local Resolution
d
Fouier Shell Correlation
FSC = 0.143
2.88
Resolution (1/Å)
0
0.1
0.2
0.3
0.4
0.5
0.2
0.4
0.6
0.8
1.0

e

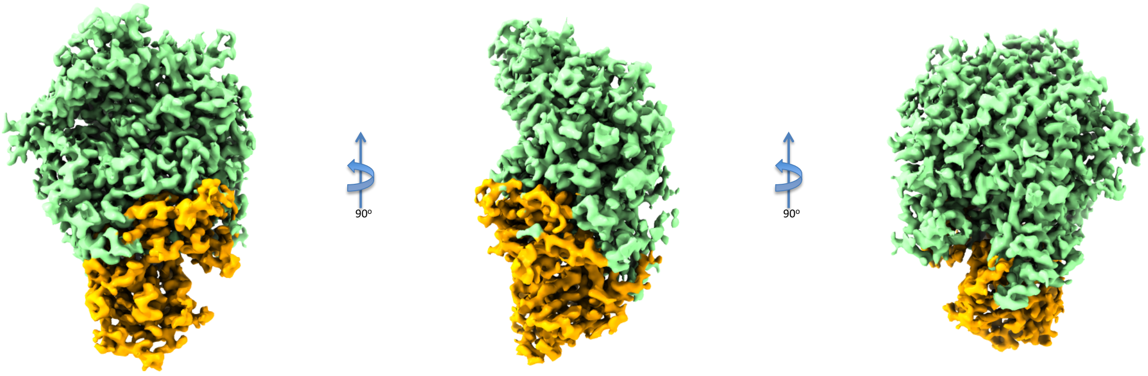

Supplementary Figure 2. Flow chart of cryo-EM image processing of P1

a) Representative microscope image and power spectra from the image. White arrow shows diffraction pattern derived from GO. **b)** 2D classification image of P1. **c)** Final 3D reconstruction map color-coded according to local resolution. **d)** Gold-standard Fourier Shell Correlation (FSC) curve of final map. **e)** Three 90° apart views of the cryo-EM map.

a

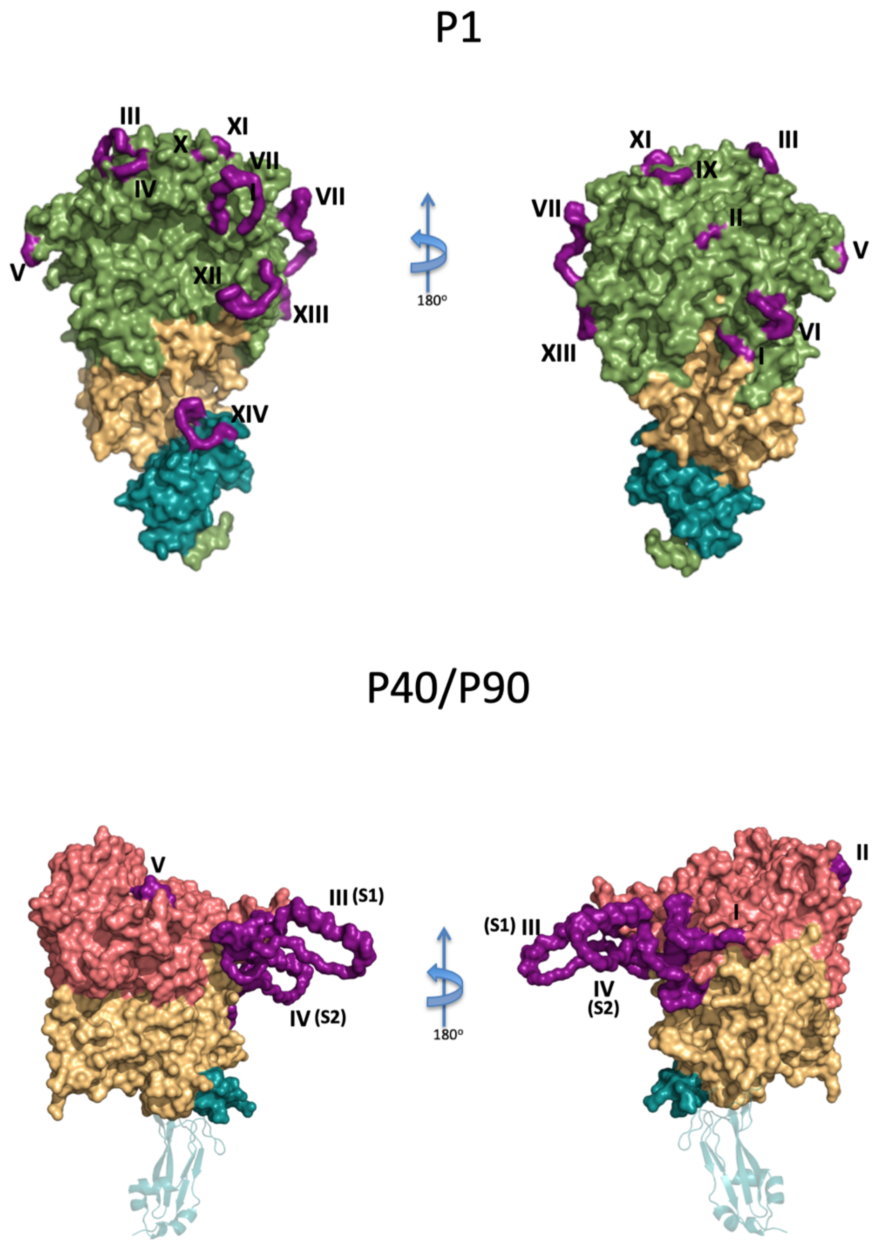
P1
III
XI
X
VII
IV
VII
V
XII
XIII
XIV
180°
XI
III
IX
VII
II
V
XIII
VI
I
P40/P90
V
III (S1)
IV (S2)
180°
II
(S1) III
I
IV
(S2)

b

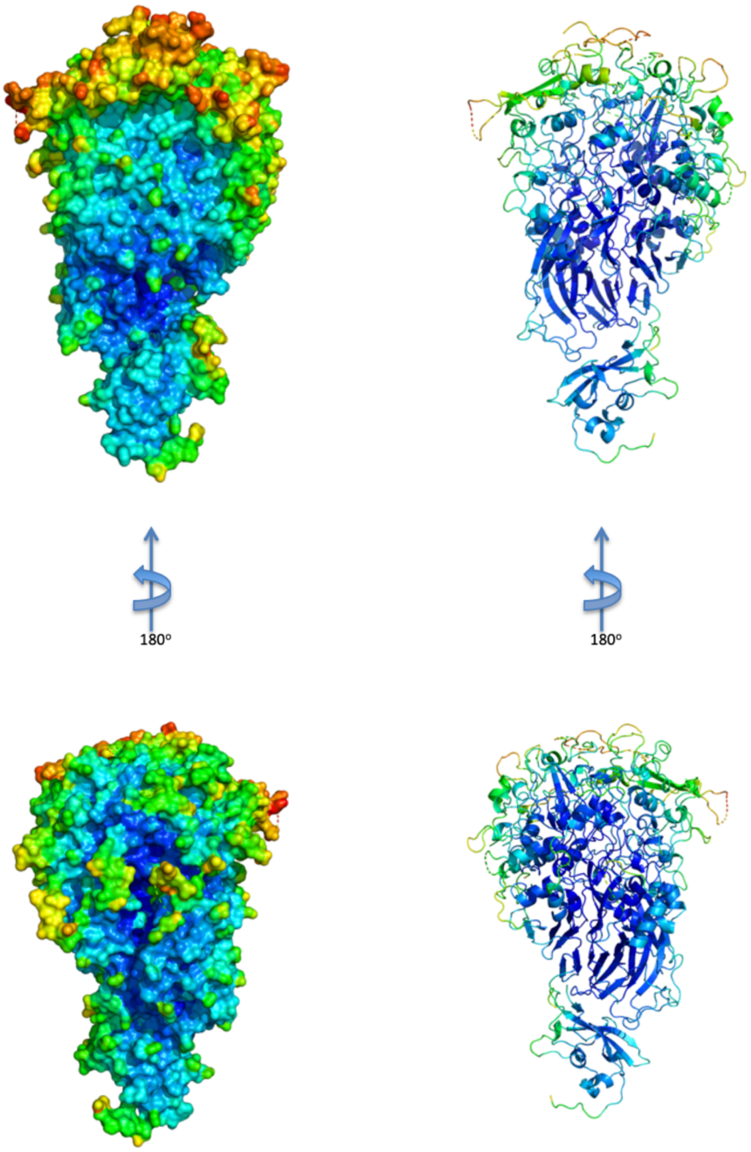

Supplementary Figure 3. Disordered regions in the structures of P1 and P40/P90

a) Protein surface representation indicating the disordered regions (purple) found in the crystal structures of P1 and P40/P90 (top and bottom panels, respectively). Most of these disordered regions correspond to insertions in the sequence with respect to the orthologue proteins P140 and P110 from *M. genitalium*. Only one disordered region, numbered XIV, is found in the C-terminal domain of P1 (cyan), while the crown of P1 contains thirteen

disordered regions, each with just a few residues. In P40/P90 there are only five disordered regions, but two of them, named as S1 and S2 (see in the text) with more than sixty residues each. There are no disordered regions in the β-propellers (brown). The disordered loops depicted in this figure, only for illustration purposes, have been built using the Sloop database [2]. **b)** Two 180° apart views of the temperature factors in the crystal structure of P1. The warmest places (red) are located in the upper part of the crown, while the coldest (dark blue) are in the β-propeller. The C-terminal domain is quite cold, indicating low mobility, contrary to what is found in the cryo-EM structure of P1.

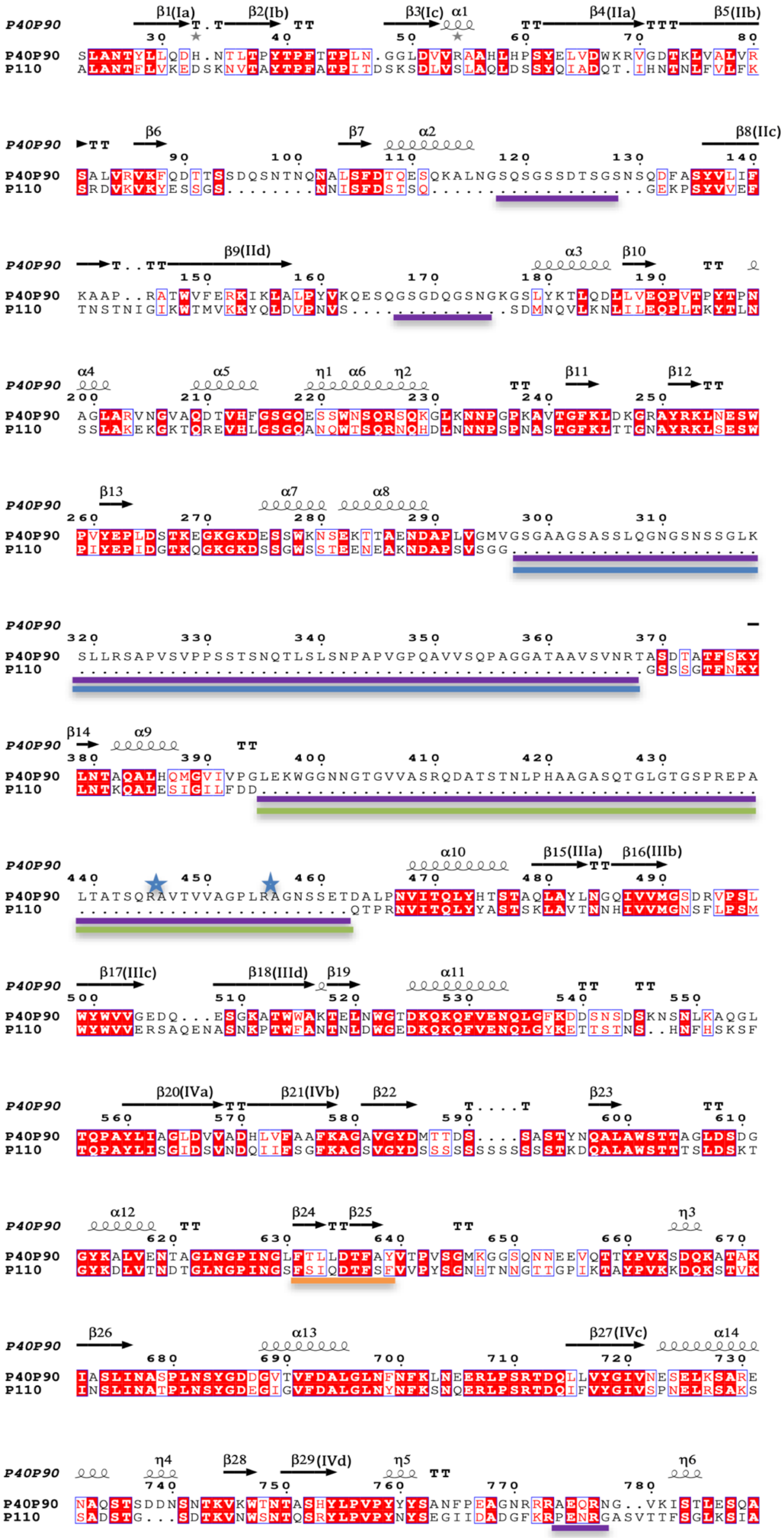

P40P90 SLANTYLLQDH.NTLTPYTPFTTPLN.GGLDVVRAAHLHPSYELVDWKRVGDTKLVALVR
P110 ALANTFLVKEDSKNVTAYTPFATPITDSKSDLVSLAQLDSSYQIADQT.IHNTNLFVLFK
P40P90 SALVRVKFQDTTSSDQSNTNQNALSFDTQESQKALNGSQSGSSDTSGSNSQDFASYVLIF
P110 SRDVKVKYESSGS........NNISFDSTSQ..................GEKPSYVVEF
P40P90 KAAP..RATWVFERKIKLALPYVKQESQGSGDQGSNGKGSLYKTLQDLLVEQPVTPYTPN
P110 TNSTNIGIKWTMVKKYQLDVPNVS...........SDMNQVLKNLILEQPLTKYTLN
P40P90 AGLARVNGVAQDTVHFGSGQESSWNSQRSQKGLKNNPGPKAVTGFKLDKGRAYRKLNESW
P110 SSLAKEKGKTQREVHLGSGQANQWTSQRNQHDLNNNPSPNASTGFKLTTGNAYRKLSESW
P40P90 PVYEPLDSTKEGKGKDESSWKNSEKTTAENDAPLVGMVGSGAAGSASSLQGNGSNSSGLK
P110 PIYEPIDGTKQGKGKDSSGWSSTEENEAKNDAPSVSGG
P40P90 SLLRSAPVSVPPSSTSNQTLSLSNPAPVGPQAVVSQPAGGATAAVSVNRTASDTATFSKY
P110 GSSSGTFNKY
P40P90 LNTAQALHQMGVIVPGLEKWGGNNGTGVVASRQDATSTNLPHAAGASQTGLGTGSPREPA
P110 LNTKQALESIGILFDD
P40P90 LTATSQRAVTVVAGPLRAGNSSETDALPNVITQLYHTSTAQLAYLNGQIVVMGSDRVPSL
P110 QTPRNVITQLYYASTSKLAVTNNHIVVMGNSFLPSM
P40P90 WYWVVGEDQ...ESGKATWWAKTELNWGTDKQKQFVENQLGFKDDSNSDSKNSNLKAQGL
P110 WYWVVERSAQENASNKPTWFANTNLDWGEDKQKQFVENQLGYKETTSTNS..HNFHSKSF
P40P90 TQPAYLIAGLDVVADHLVFAAFKAGAVGYDMTTDS....SASTYNQALAWSTTAGLDSDG
P110 TQPAYLISGIDSVNDQIIFSGFKAGSVGYDSSSSSSSSSSTKDQALAWSTTTSLDSKT
P40P90 GYKALVENTAGLNGPINGLFTLLDTFAYVTPVSGMKGGSQNNEEVQTTYPVKSDQKATAK
P110 GYKDLVTNDTGLNGPINGSFSIQDTFSFVVPYSGNHTNNGTTGPIKTAYPVKKDQKSTVK
P40P90 IASLINASPLNSYGDDGVTVFDALGLNFNFKLNEERLPSRTDQLLVYGIVNESELKSARE
P110 INSLINATPLNSYGDEGIGVFDALGLNYNFKSNQERLPSRTDQIFVYGIVSPNELRSAKS
P40P90 NAQSTSDDNSNTKVKWTNTASHYLPVPYYSANFPEAGNRRAEQRNG..VKISTLESQA
P110 SADSTG...SDTKVNWSNTQSRYLPVPYNYSEGIIDADGFKRPENRGASVTTFSGLKSIA

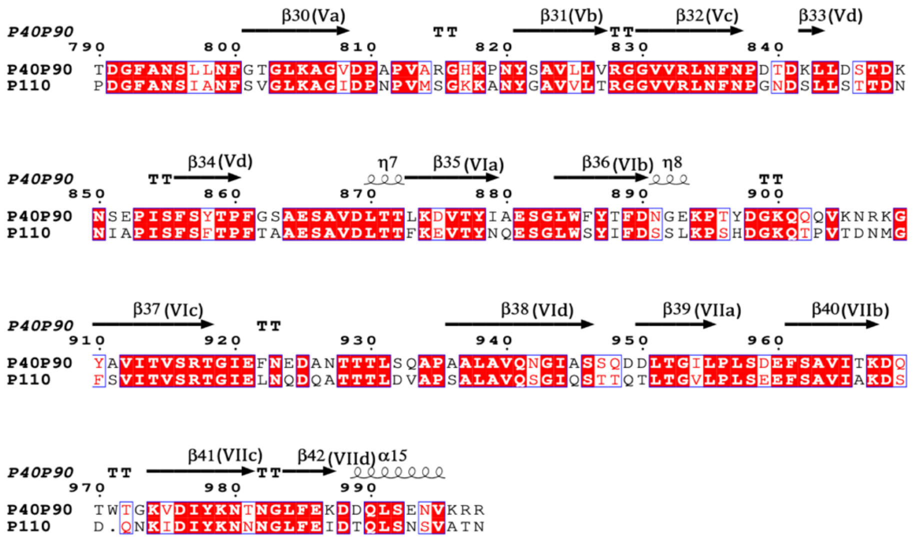

Supplementary Figure 4. **Structural sequence alignments of P40/P90**

Structural sequence alignments obtained by the superposition between P40/P90 and the orthologous protein P110 from *M. genitalium* and represented with program Espript [1]. The DSSP assignment of secondary structure elements for P40/P90 are represented above the sequence, as squiggles (α-helices), arrows (β-strands) and TT (turns). Missing residues, corresponding to disordered regions of P40/P90 in the X-ray structure, are indicated with a purple line. The long insertions 1 and 2 (named as S1 and S2) are underlined in blue and green, respectively. An orange line indicates the sialic acid oligosaccharide binding loop and the two blue stars correspond to the Arg445-Ala446 and Arg455-Ala456 cleavage sites.

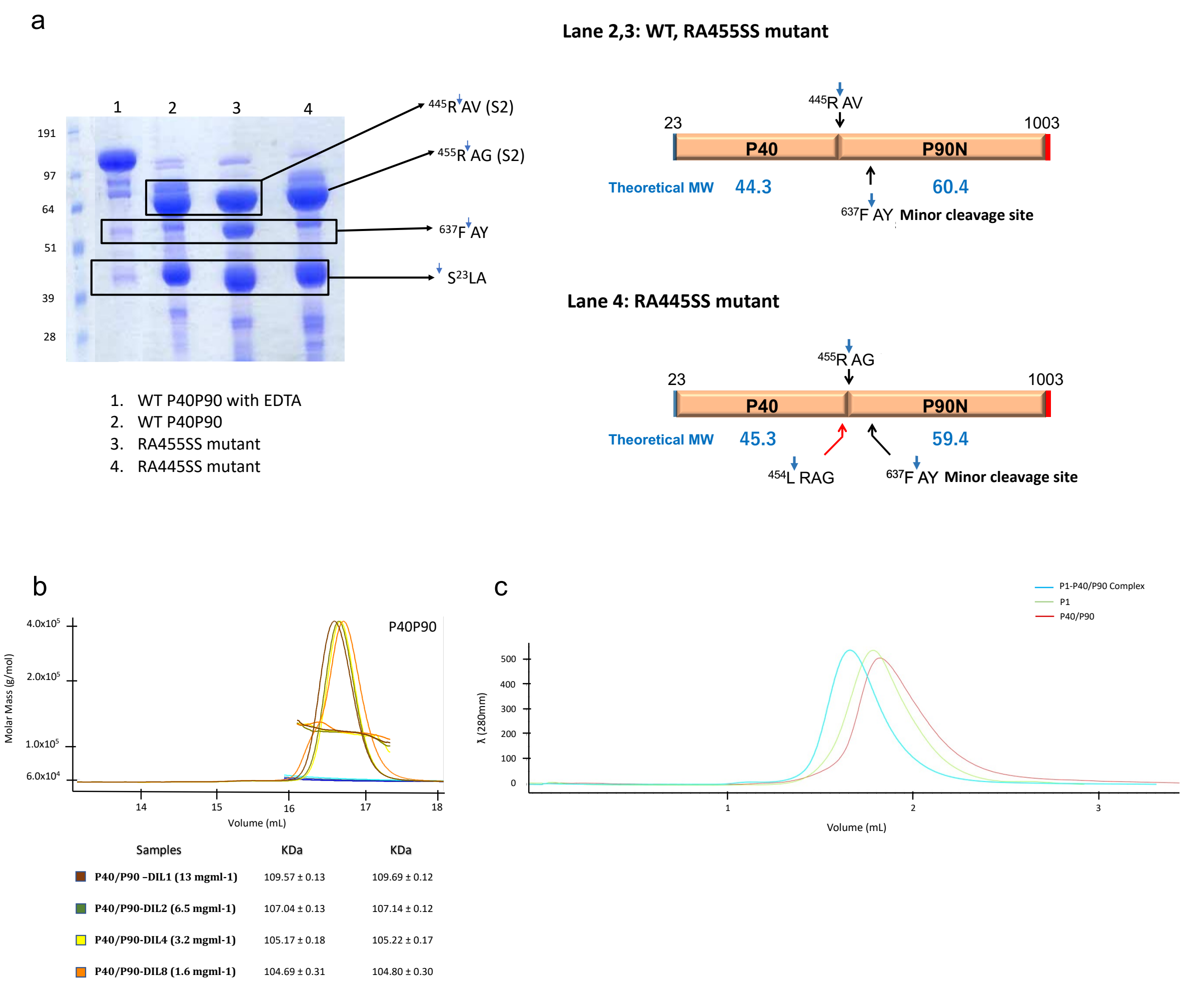

Supplementary Figure 5. Purification and cleavages of P40/P90

a) SDS-PAGE of purified samples from the P40/P90 ectodomain (residues from Ser23 to Pro1113) always showed several bands (left panel). For the construct with the wild type (WT) sequence and in the presence of EDTA (lane 1) the dominant band corresponds to the full construct of ~105 kDa. The pattern changed completely in the absence of EDTA (lane 2), with the band corresponding to the full construct almost disappearing and two new strong bands corresponding to polypeptides starting at Ser23 and Ala446 and with molecular weights of about 45 and 60 kDa, respectively. In the absence of EDTA the degradation pattern remains similar for the P40/P90 variant where the two residues from the cleavage motif (Arg445-Ala446) were replaced by serines (lane 4). However, now the band corresponding to the large polypeptide starts with Ala456. Finally, the pattern is

again very similar to the wild type for the P40/P90 variant where the two residues of the second RA cleavage motif Arg455-Ala456 were replaced by serines (lane 3). The relative size of the small and large polypeptides (right panel) could well correspond to the P40 and P90 subunits if the trans-membrane and cytoplasmic regions are added to the large polypeptide. Previously described cleavage site ^{454}LRAG is also shown [3] **b)** Analysis by MALS of purified samples of P40/P90N detected only one monomeric specie with a molecular weight of ~107 kDa**. c)** Chromatographic profiles (with a Superdex 200 5/150) of P1 (green), P40/P90 (red) and the P1-P40/P90 heterodimer (light blue).

a

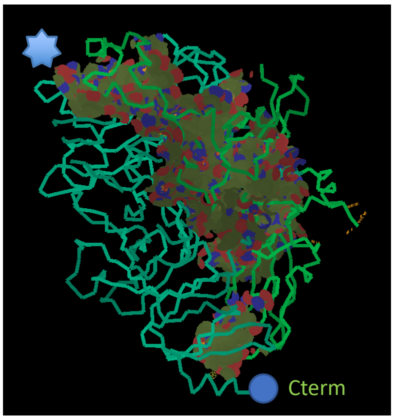

b

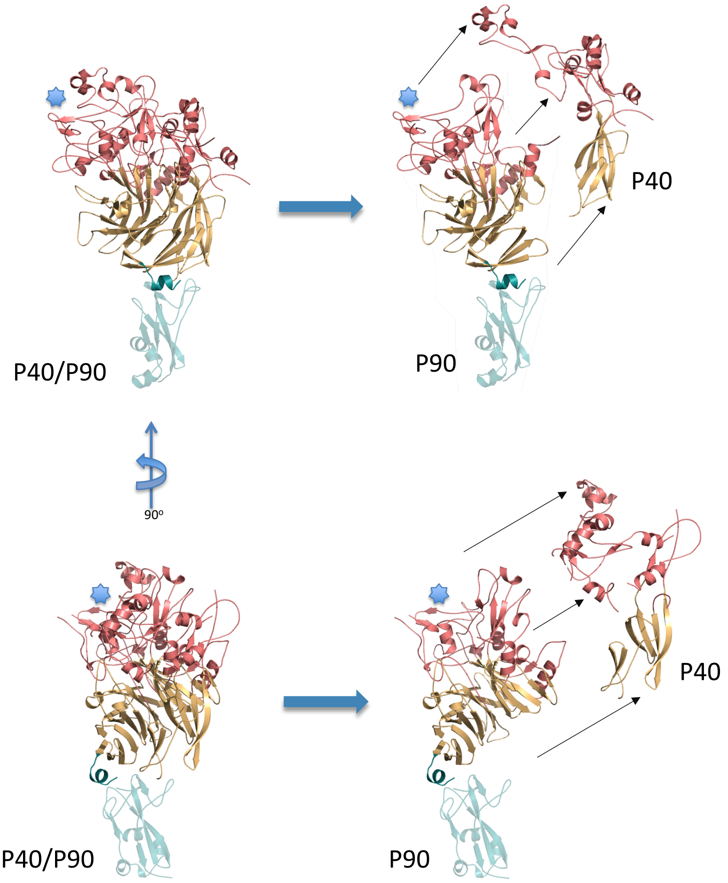

Supplementary Figure 6. P40 and P90 polypeptides in P40/P90

a) The P40 polypeptide (solid representation) sits on the surface of the P90 polypeptide, with a large interacting surface. The sialic acid binding site is indicated with a blue star. Colored blobs indicate interface contacts. **b)** Separation of the two polypeptides would

divide the β-propeller and alter the sialic binding site, although all the residues interacting directly with the neuraminic moiety belong to the P90 polypeptide.

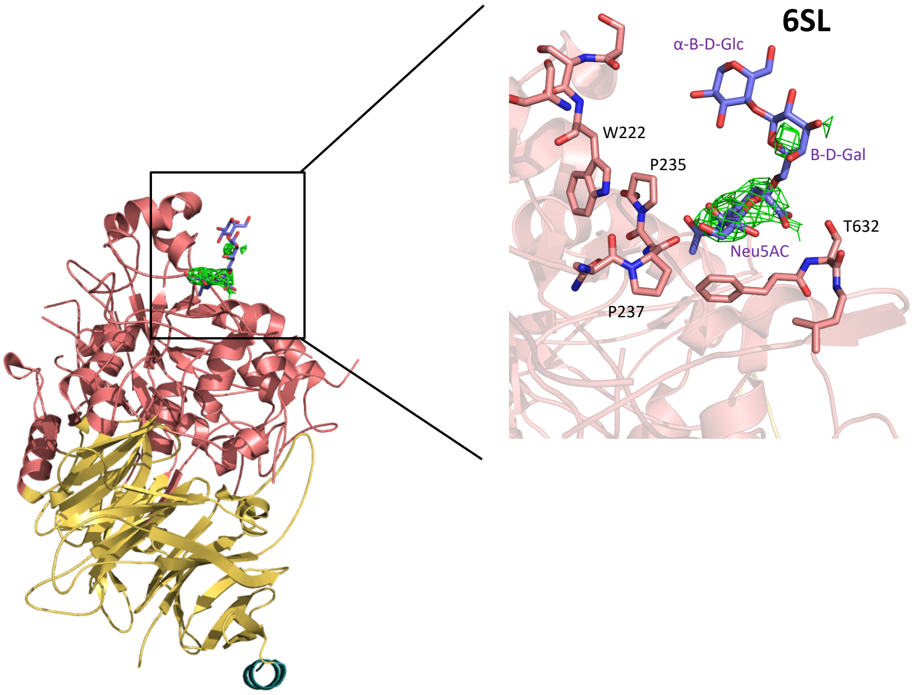

Supplementary Figure 7. Binding of oligosaccharide 6SL to P40/P90

Ribbon representation of the P40/P90N structure in complex with oligosaccharide 6SL. The inset shows a detail of the residues shaping the binding pocket. The electron density omit map (at 0.9 sigma) corresponding to 6SL is also shown.

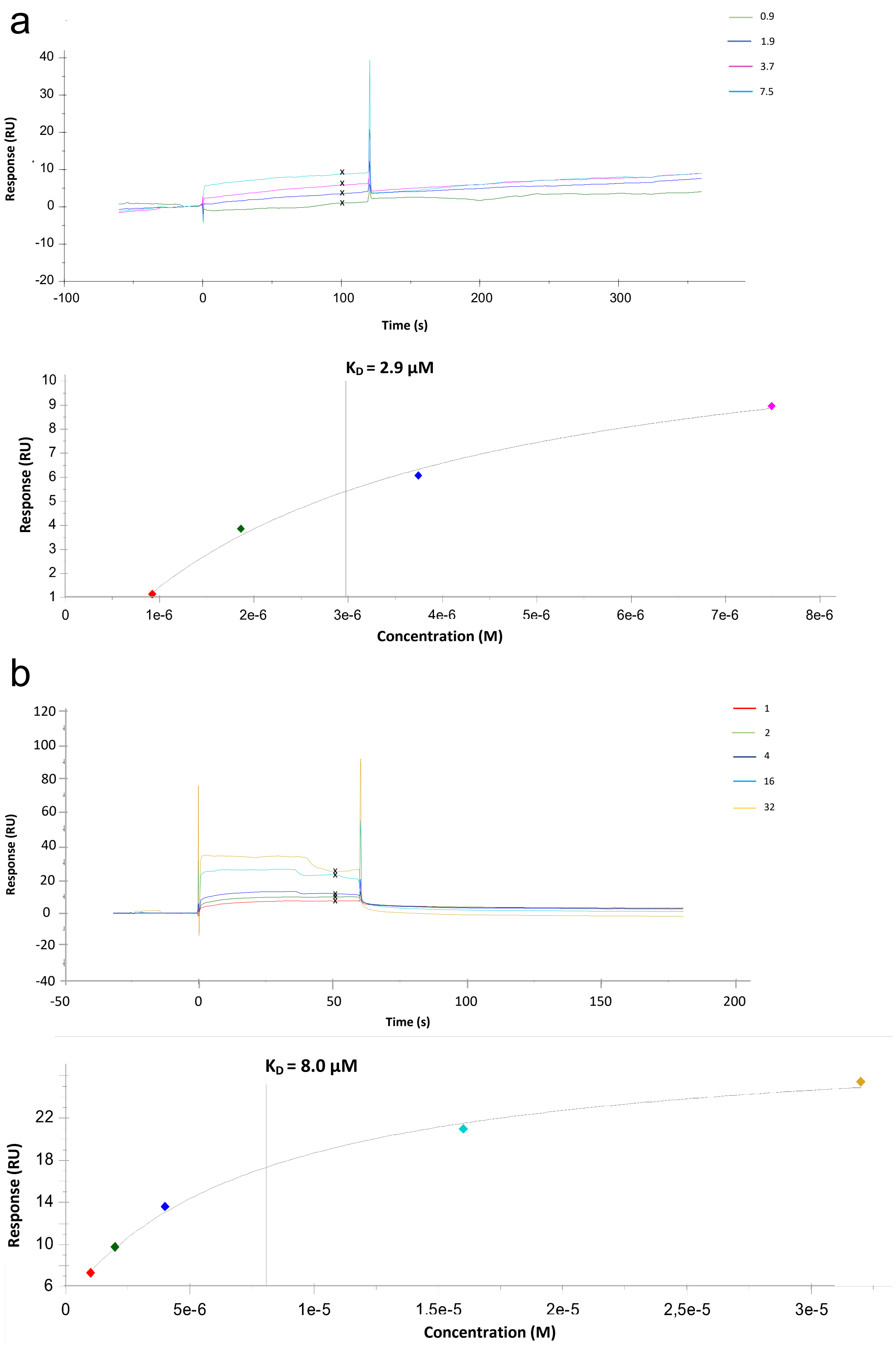
a
0.9
1.9
3.7
7.5
Response (RU)
Time (s)
K$_D$ = 2.9 µM
Response (RU)
Concentration (M)
b
1
2
4
16
32
Response (RU)
Time (s)
K$_D$ = 8.0 µM
Response (RU)
Concentration (M)

c

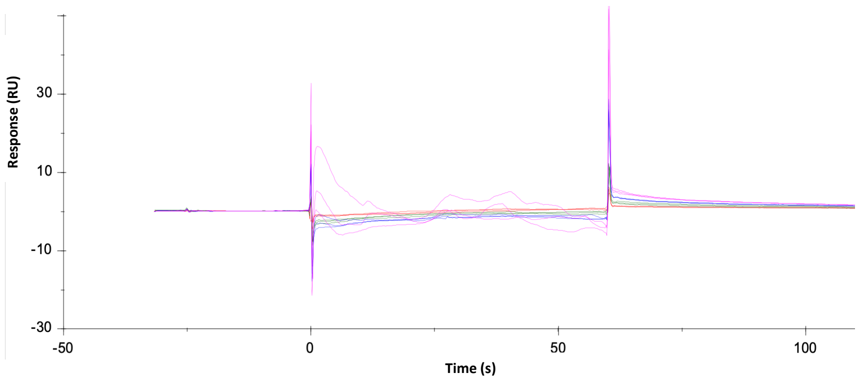

Supplementary Figure 8. Binding of oligosaccharides 3SL and 6SL to P40/P90

Sensorgrams (upper panels) and affinity plots (lower panels), obtained by Surface Plasmon Resonance (SPR), for the binding to protein P40/P90 of **a)** 6SL **b)** and 3SL. **c)** For protein P1 no binding was detected with 3SL. Source data are provided as a Source Data file.

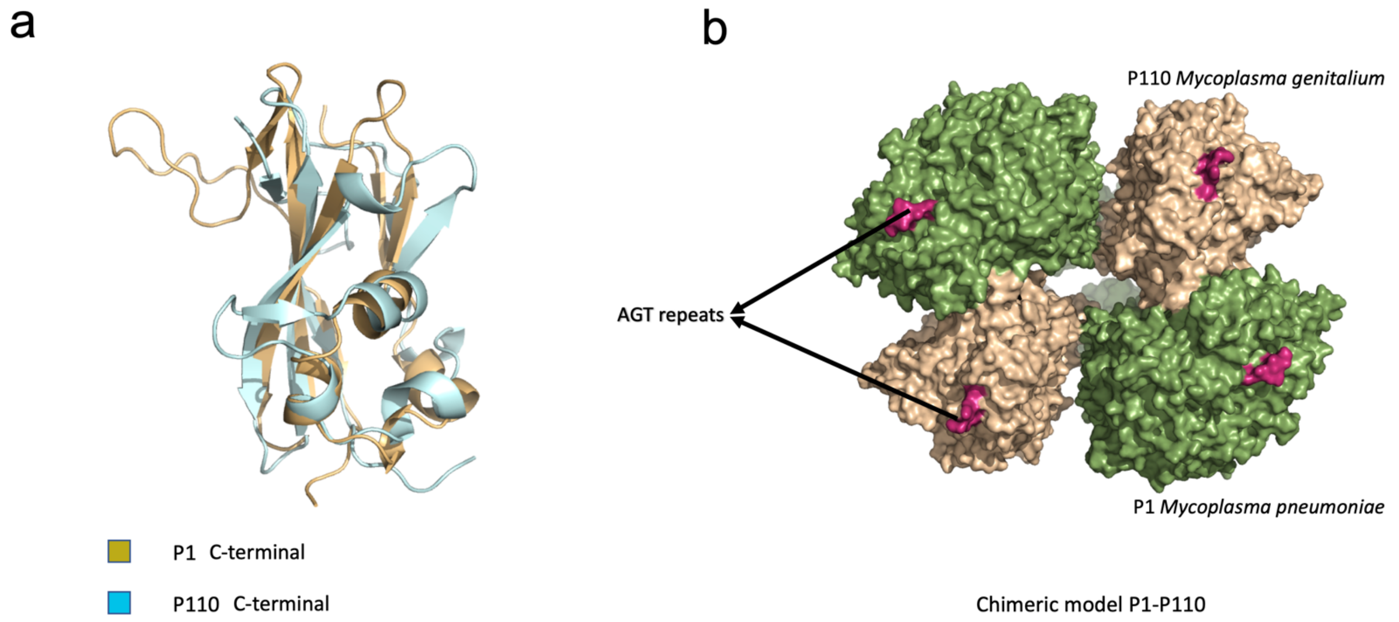

Supplementary Figure 9. Structural relationships between P1 and P110

a) Superposition of the C-terminal domains from P1 and from the *M.genitalium* non-orthologous adhesin P110. **b)** Modelling of a chimeric Nap with P1 and P110, from the structure determined in *M. genitalium*. AGT repeats are colored in warm pink. Similarities between the two structures reinforces the idea of a common ancestor and the possibility of a four-fold symmetry organization in the Nap precursor.

>P1_60-1518

mAb-aN
NAINPRLTPWTYRNTSFSSLPLTGENPGAWALVRDNSAKGITAGSGSQQTTYDPTRTEAALTASTTFALRRYDLAGRALYDLDFSKLN

PQTPTRDQTGQITFNPFGGFGLSGAAPQQWNEVKNKVPVEVAQDPSNPYRFAVLLVPRSVVYYEQLQRGLGLPQQRTESGQNTS

mAb-P1.62
TTGAMFGLKVKNAEADTAKSNEKLQGAEATGSSTTSGSGQSTQRGGSSGDTKVKALKIEVKKKSDSEDNGQLQLEKNDLANAPIKR

SEESGQSVQLKADDFGTALSSSGSGGNSNPGSPTPWRPWLATEQIHKDLPKWSASILILYDAPYARNRTAIDRVDHLDPKAMTANY

PPSWRTPKWNHHGLWDWKARDVLLQTTGFFNPRRHPEWFDGGQTVADNEKTGFDVDNSENTKQGFQKEADSDKSAPIALPFE

AYFANIGNLTWFGQALLVFGGNGHVTKSAHTAPLSIGVFRVRYNATGTSATVTGWPYALLFSGMVNKQTDGLKDLPFNNNRWFEY

VPRMAVAGAKFVGRELVLAGTITMGDTATVPRLLYDELESNLNLVAQGQGLLREDLQLFTPYGWANRPDLPIGAWSSSSSSSHNAP

YYFHNNPDWQDRPIQNVVDAFIKPWEDKNGKDDAKYIYPYRYSGMWAWQVYNWSNKLTDQPLSADFVNENAYQPNSLFAAIL

Patient's sera
NPELLAALPDKVKYGKENEFAANEYERFNQKLTVAPTQGTNWSHFSPTLSRFSTGFNLVGSVLDQVLDYVPWIGNGYRYGNNHRG

VDDITAPQTSAGSSSGISTNTSGSRSFLPTFSNIGVGLKANVQATLGGSQTMITGGSPRRTLDQANLQLWTGAGWRNDKASSGQS

mAb-P1.62 mAb-P1.26
DENHTKFTSATGMDQQGQSGTSAGNPDSLKQDNISKSGDSLTTQDGNAIDQQEATNYTNLPPNLTPTADWPNALSFTNKNNAQ

RAQLFLRGLLGSIPVLVNRSGSDSNKFQATDQKWSYTDLHSDQTKLNLPAYGEVNGLLNPALVETYFGNTRAGGSGSNTTSSPGIGF

Patient's sera
KIPEQNNDSKATLITPGLAWTPQDVGNLVVSGTTVSFQLGGWLVTFTDFVKPRAGYLGLQLTGLDASDATQRALIWAPRPWAAFR

GSWVNRLGRVESVWDLKGVWADQAQSDSQGSTTTATRNALPEHPNALAFQVSVVEASAYKPNTSSGQTQSTNSSPYLHLVKPKK

VTQSDKLDDDLKNLLDPNQVRTKLRQSFGTDHSTQPQPQSLKTTTPVFGTSSGNLSSVLSGGGAGGGSSGSGQSGVDLSPVEKVS

mAb-P1.26 mAb-6E7
GWLVGQLPSTSDGNTSSTNNLAPNTNTGNDVVGVGRLSESNAAKMNDDVDGIVRTPLAELLDGEGQTADTGPQSVKFKSPDQID

mAb-M58 mAb-M51
FNRLFTHPVTDLFDPVTMLVYDQYIPLFIDIPASVNPKMVRLKVLSFDTNEQSLGLRLEFFKPDQDTQPNNNVQVNPNNGDFLPLLT

ASSQGPQTLFSPFNQ

Adherence inhibiting monoclonal antibodies

mAb	Epitopes		References
aN	NAINPRLTPWTYRN		
P1.26	NALSFTNK	DVVGVGRL	
P1.62	EVKKKSDS	ENHTKFTS	5
M58	SVNPKMVR		
M51	NEQSLGLR		
6E7	GIVRTPLAELLDG		4

Patient's sera

	Imunodominant epitopes	Reference
Patient's sera	WIGNGYRY<br>FTDFVKPR	6

Supplementary Figure 10. Reported epitopes for P1

Mapping of epitopes was generated using data from references [4-6]

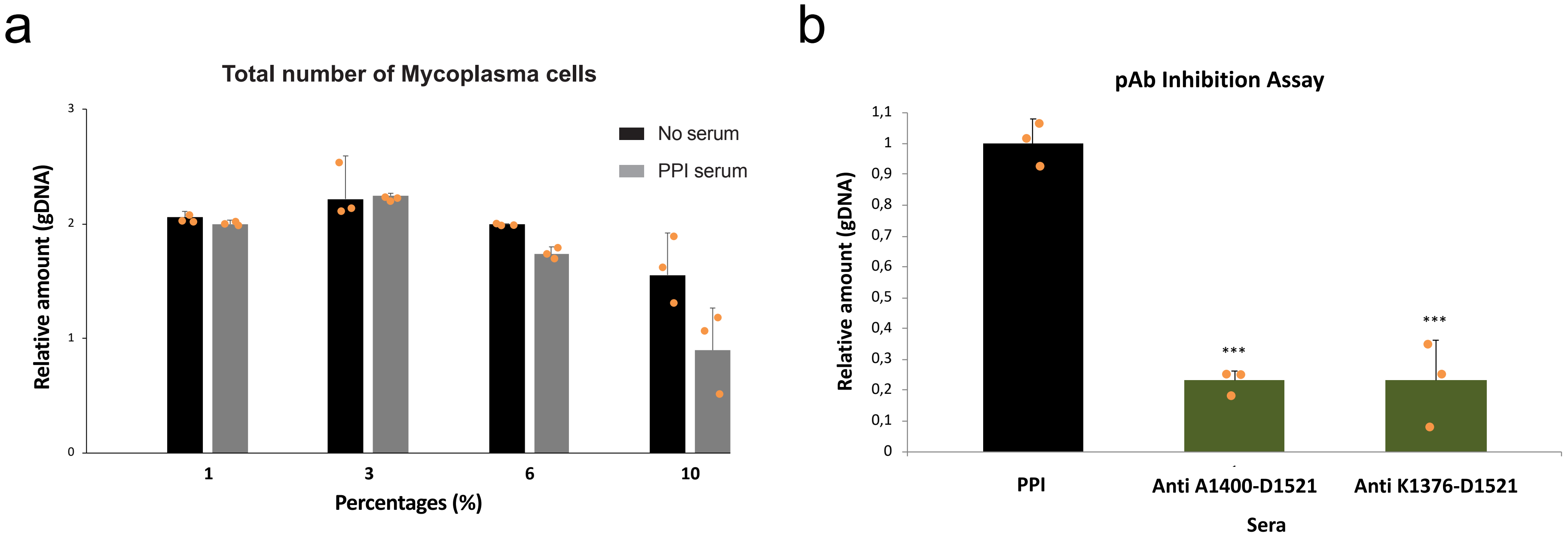

Supplementary Figure 11. Toxicity and adhesion inhibition of Mycoplasma cells

a) Relative amount of genomic DNA from *M. pneumoniae* with respect to the increasing percentages of PPI serum. No serum is the control value for non-toxicity. Sera concentrations of 6% show some toxicity that increases at 10%. **b)** Binding inhibition observed in cells when incubated with 10% serum generated against the P1 C-terminal domain short and long constructs Ala1400-Asp1521 and Lys1376-Asp1521, respectively. Results have been normalized with PPI value to minimize the effect of toxicity. Error bars represent the mean ± standard deviation. *** $p < 0{,}001$. Source data are provided as a Source Data file.

Supplementary Table 1. X-ray Data collection and Refinement statistics

Data collection[(a)]	P1	P40/P90	P40P90-3SL	P40P90-6SL
**Space Group**	C 1 2 1	P 21 21 21	C 1 2 1 *	C 1 2 1 *
**Cell dimensions**				
**a,b,c (Å)**	132.90, 115.64, 96.34	97.8,0 114.43, 165.05	116.12, 107.31, 162.03	116.33, 107.38, 160.45
**α, β, γ (º)**	90.00, 117.67, 90.00	90.00, 90.00, 90.00	90.00, 90.12, 90.00	90.00, 90.10, 90.00
**Unique reflections**	92405 (13355)	54524 (7870)	35571 (5211)	471848 (6819)
**Resolution (Å)**	72.20-1.94 (2.04-1.94)	97.8-2.65 (2.79-2.65)	81.01-3.10 (3.27-3.10)	80.22-2.80 (2.95-2.80)
**Wavelength (Å)**	0.97897	0.97927	0.97926	0.97926
**Rmeas (%)** [b]	0.143 (1.505)	0.145 (1.872)	0.149 (1.281)	0.121 (1.324)
**I/Isigma**	6.9 (1.1)	8.6 (1.0)	5.3 (1.0)	7.4 (1.0)
**CC (½)**	0.993 (0.658)	0.996 (0.621)	0.989 (0.315)	0.996 (0.252)
**Completness (%)**	96.5 (95.7)	99.8 (99.7)	97.4 (97.9)	97.0 (96.6)
**Redundancy**	3.5 (3.6)	6.5 (6.3)	3.1 (3.1)	2.9 (2.9)

Refinement statistcs[(a)]				
**Resolution**	72.20-1.94 (1.99-1.94)	82.53-2.65 (2.67-2.65)	81.01-3.10 (3.12-3.10)	80.22-2.80 (2.82-2.80)
**Num. reflections**	92369 (6370)	54428 (1044)	35571 (661)	47182 (913)
**R_{Cryst} (%)** [c]	18.7 (23.28)	21.4 (25.70)	24.40 (23.10)	20.90 (23.35)
**R_{free} (%)** [d]	22.9 (25.69)	23.4 (32.60)	27.90 (24.75)	26.50 (25.02)
**Model Composition**				
**Nº Residues** [e]	1339	1618	1613	1622
**Nº Ligands**	0	0	2	2
**Solvent content (%)**	40.0	44.4	57.4	56.6
**R.m.s deviations**				
**Bond lengths ($Å^2$)**	0.01	0.008	0.008	0.01
**Bond angles (º)**	1.15	1.08	1.03	1.19
***B* factor ($Å^2$)**				
**Protein**	60.7	89.5	147.2	96.1
**Solvent**	43.5	59.7	-	-
**Ligand**	-	-	245.0 **	116.5
**Validation**				
**MoltProbity score**	2.26	2.18	2.56	2.58
**Clashscore**	3.70	2.50	4.40	5.50
**Poor rotamers (%)**	6.23	9.50	10.20	13.00
**Ramachandran plot**				
**Favored (%)**	91.9	93.9	89.0	91.4
**Allowed (%)**	5.6	4.7	9.1	7.0
**Disallowed (%)**	2.5	1.4	1.9	1.6

[a] Values in parentheses correspond to the highest resolution Shell.

[b] $R_{sym} = \Sigma_{hkl}\Sigma_i I_i(hkl) - \langle I(hkl)\rangle / \Sigma_{hkl}\Sigma_i I_i(hkl)$, where $I_i(hkl)$ is the intensity of an observation and $\langle I(hkl)\rangle$ is the mean value of observations for an unique reflection.

[c] $R_{cryst} = \Sigma_h F_0(h) - F_c(h) / \Sigma_h F_0(h)$, where F_o and F_C are the observed and calculated structure-factors amplitudes, respectively.

[d] R_{free} was calculated with 5% of data, which was excluded from refinement.

[e] Number of residues by unit cell.

[*] P40/P90 complexes presented a pseudorthorhombic packing within a monoclinic C2 space group.

[**] *B* factor obtained after refinement with ligand occupancy 1.0. The *B* factor decreased to 142.3 $Å^2$ for a ligand occupancy of 0.7.

Supplementary Table 2. Cryo-EM Data collection and Refinement statistics

Data collection and processing	P1
Magnification	96,000
Voltage (kV)	300
Electron exposure ($e^-/Å^2$)	60
Defocus range (µm)	-0.75 to -2.75
Pixel size (Å)	0,69
Symmetry imposed	C1
Initial particle images (nº)	4,574,424
Final particle images (nº)	68,014
Map Resolution	2.88
FSC threshold	0.143
Map resolution range (Å)	2.8 to 4.0
**Refinement statistcs**	
Initial model used	6RC9 (X-ray structure)
Model resolution (Å)	2.94
FSC threshold	0.5
Model resolution range (Å)	2.8 to 4.0
Map sharpening *B* factor ($Å^2$)	-89.4
Model composition	
Nonhydrogen atoms	9532
Protein residues	1219
Nº Ligands	-
B factor ($Å^2$)	59.175
R.m.s deviations	
Bond lengths ($Å^2$)	0.005
Bond angles (º)	0.769
Validation	
MoltProbity score	2.45
Clashscore	7.37
Poor rotamer (%)	7.02
Ramachandran plot	
Favored (%)	93.96
Allowed (%)	6.04
Disallowed (%)	0.00

Supplementary Table 3. Synthetic DNA for P1

ATGCACCAGACCAAGAAAACCGCGCTGAGCAAGAGCACCTGGATCCTGATTCTGACCGCGACCGCGAGCCTGGCGACCGGTCTGACCGTGGTTGGTCACTTTACCAGCACCACCACCACC
CTGAAACGTCAGCAATTCAGCTATACCCGTCCGGACGAAGTTGCGCTGCGTCACACCAACGCGATCAACCCGCGTCTGACCCCGTGGACCTACCGTAACACCAGCTTCAGCAGCCTGCCG
CTGACCGGCGAGAACCCGGGTGCGTGGGCGCTGGTGCGTGACAACAGCGCGAAGGGTATTACCGCGGGTAGCGGCAGCCAGCAAACCACCTATGATCCGACCCGTACCGAAGCGGCGCT
GACCGCGAGCACCACCTTTGCGCTGCGTCGTTACGACCTGGCGGGTCGTGCGCTGTATGATCTGGACTTCAGCAAACTGAACCCGCAGACCCCGACCCGTGATCAAACCGGTCAGATCAC
CTTCAACCCGTTTGGTGGCTTCGGTCTGAGCGGTGCGGCGCCGCAGCAATGGAACGAGGTTAAAAACAAGGTGCCGGTTGAAGTGGCGCAAGACCCGAGCAACCCGTACCGTTTTGCGGT
TCTGCTGGTGCCGCGTAGCGTGGTTTACTATGAACAGCTGCAACGTGGTCTGGGCCTGCCGCAGCAACGTACCGAGAGCGGTCAGAACACCAGCACCACCGGTGCGATGTTCGGCCTGAA
AGTGAAGAACGCGGAAGCGGATACCGCGAAAAGCAACGAGAAGCTGCAGGGTGCGGAAGCGACCGGTAGCAGCACCACCAGCGGTAGCGGTCAAAGCACCCAGCGTGGTGGCAGCAGC
GGTGACACCAAAGTTAAGGCGCTGAAGATCGAGGTGAAGAAAAAGAGCGATAGCGAAGACAACGGCCAGCTGCAACTGGAGAAAAACGATCTGGCGAACGCGCCGATTAAGCGTAGCG
AGGAAAGCGGTCAAAGCGTTCAGCTGAAAGCGGACGATTTTGGTACCGCGCTGAGCAGCAGCGGTAGCGGTGGCAACAGCAACCCGGGTAGCCCGACCCCGTGGCGTCCGTGGCTGGCG
ACCGAGCAGATCCACAAAGACCTGCCGAAGTGGAGCGCGAGCATCCTGATTCTGTACGATGCGCCGTATGCGCGTAACCGTACCGCGATTGATCGTGTTGACCACCTGGATCCGAAGGCG
ATGACCGCGAACTACCCGCCGAGCTGGCGTACCCCGAAATGGAACCACCACGGTCTGTGGGACTGGAAGGCGCGTGATGTGCTGCTGCAGACCACCGGCTTCTTTAACCCGCGTCGTCAC
CCGGAATGGTTTGACGGTGGCCAAACCGTTGCGGATAACGAGAAGACCGGTTTTGATGTGGACAACAGCGAAAACACCAAACAAGGCTTCCAGAAAGAGGCGGATAGCGACAAAAGCG
CGCCGATTGCGCTGCCGTTTGAGGCGTATTTCGCGAACATTGGTAACCTGACCTGGTTTGGCCAGGCGCTGCTGGTTTTCGGTGGCAACGGTCATGTTACCAAGAGCGCGCACACCGCGCC
GCTGAGCATTGGTGTTTTTCGTGTGCGTTACAACGCGACCGGTACCAGCGCGACCGTTACCGGCTGGCCGTATGCGCTGCTGTTCAGCGGTATGGTGAACAAACAGACCGACGGCCTGAA
GGATCTGCCGTTTAACAACAACCGTTGGTTCGAATACGTTCCGCGTATGGCGGTGGCGGGTGCGAAATTTGTTGGCCGTGAGCTGGTGCTGGCGGGTACCATTACCATGGGTGACACCGCG
ACCGTTCCGCGTCTGCTGTATGATGAGCTGGAAAGCAACCTGAACCTGGTGGCGCAGGGTCAAGGCCTGCTGCGTGAAGACCTGCAGCTGTTCACCCCGTACGGTTGGGCGAACCGTCCG
GATCTGCCGATTGGCGCGTGGAGCTCTTCTAGCAGCAGCAGCCACAACGCGCCGTACTATTTTCACAACAACCCGGACTGGCAAGATCGTCCGATCCAGAACGTGGTTGACGCGTTCATTA
AACCGTGGGAGGATAAAAACGGCAAGGACGATGCGAAGTACATCTATCCGTACCGTTATAGCGGCATGTGGGCGTGGCAAGTTTATAACTGGAGCAACAAGCTGACCGACCAGCCGCTG
AGCGCGGATTTTGTGAACGAAAACGCGTACCAGCCGAACAGCCTGTTCGCGGCGATTCTGAACCCGGAGCTGCTGGCGGCGCTGCCGGACAAGGTTAAGTACGGCAAAGAGAACGAATT
TGCGGCGAACGAGTATGAACGTTTCAACCAAAAGCTGACCGTGGCGCCGACCCAGGGTACCAACTGGAGCCACTTCAGCCCGACCCTGAGCCGTTTTAGCACCGGTTTCAACCTGGTTGG
CAGCGTGCTGGACCAGGTTCTGGATTATGTGCCGTGGATCGGTAACGGCTACCGTTATGGTAACAACCACCGTGGCGTGGACGATATTACCGCGCCGCAAACCAGCGCGGGTAGCAGCAG
CGGCATTAGCACCAACACCAGCGGTAGCCGTAGCTTTCTGCCGACCTTCAGCAACATCGGTGTTGGCCTGAAAGCGAACGTGCAAGCGACCCTGGGTGGCAGCCAGACCATGATTACCGG
TGGCAGCCCGCGTCGTACCCTGGACCAAGCGAACCTGCAGCTGTGGACCGGTGCGGGCTGGCGTAACGACAAAGCGAGCAGCGGTCAGAGCGATGAAAACCACACCAAGTTTACCAGCG
CGACCGGTATGGACCAGCAGGGTCAGAGCGGTACCAGCGCGGGTAACCCGGACAGCCTGAAACAGGATAACATCAGCAAGAGCGGTGATAGCCTGACCACCCAAGACGGCAACGCGAT
TGATCAGCAAGAGGCGACCAACTACACCAACCTGCCGCCGAACCTGACCCCGACCGCGGACTGGCCGAACGCGCTGAGCTTTACCAACAAGAACAACGCGCAACGTGCGCAGCTGTTCCT
GCGTGGTCTGCTGGGCAGCATCCCGGTTCTGGTGAACCGTAGCGGTAGCGACAGCAACAAATTCCAAGCGACCGATCAAAAGTGGAGCTACACCGACCTGCACAGCGATCAGACCAAAC
TGAACCTGCCGGCGTATGGTGAAGTTAACGGCCTGCTGAACCCGGCGCTGGTTGAAACCTACTTTGGTAACACCCGTGCGGGTGGCAGCGGTAGCAACACCACCAGCAGCCCGGGTATCG
GCTTCAAAATTCCGGAACAGAATAACGACAGCAAAGCGACCCTGATTACCCCGGGCCTGGCGTGGACCCCGCAGGATGTTGGTAACCTGGTGGTTAGCGGCACCACCGTGAGCTTTCAAC
TGGGTGGCTGGCTGGTTACCTTTACCGACTTCGTGAAGCCGCGTGCGGGTTACCTGGGCCTGCAACTGACCGGTCTGGATGCGAGCGATGCGACCCAACGTGCGCTGATTTGGGCGCCGCG
TCCGTGGGCGGCGTTCCGTGGTAGCTGGGTTAACCGTCTGGGCCGTGTTGAGAGCGTGTGGGATCTGAAAGGTGTGTGGGCGGACCAGGCGCAAAGCGATAGCCAAGGTAGCACCACCAC
CGCGACCCGTAACGCGCTGCCGGAACACCCGAACGCGCTGGCGTTCCAAGTTAGCGTGGTTGAGGCGAGCGCGTATAAACCGAACACCAGCAGCGGTCAGACCCAAAGCACCAACAGCA
GCCCGTACCTGCACCTGGTTAAGCCGAAAAAGGTGACCCAGAGCGACAAACTGGACGATGACCTGAAGAACCTGCTGGATCCGAACCAAGTGCGTACCAAACTGCGTCAGAGCTTTGGT
ACCGACCACAGCACCCAGCCGCAACCGCAGAGCCTGAAGACCACCACCCCGGTTTTCGGTACCAGCAGCGGCAACCTGAGCAGCGTGCTGAGCGGTGGCGGTGCGGGCGGTGGCAGCAG
CGGTAGCGGCCAGAGCGGTGTTGATCTGAGCCCGGTTGAAAAGGTGAGCGGTTGGCTGGTTGGTCAACTGCCGAGCACCAGCGACGGTAACACCAGCAGCACCAACAACCTGGCGCCGA
ACACCAACACCGGCAACGATGTGGTTGGTGTTGGCCGTCTGAGCGAGAGCAACGCGGCGAAAATGAACGATGACGTTGACGGTATCGTTCGTACCCCGCTGGCGGAGCTGCTGGATGGTG
AGGGTCAGACCGCGGATACCGGTCCGCAGAGCGTTAAATTCAAGAGCCCGGACCAGATCGATTTTAACCGTCTGTTCACCCACCCGGTGACCGACCTGTTTGATCCGGTTACCATGCTGGT
GTACGACCAATATATTCCGCTGTTCATCGATATTCCGGCGAGCGTGAACCCGAAAATGGTTCGTCTGAAGGTGCTGAGCTTTGACACCAACGAACAGAGCCTGGGTCTGCGTCTGGAGTTC
TTTAAACCGGATCAAGACACCCAGCCGAACAACAACGTTCAAGTGAACCCGAACAACGGTGACTTCCTGCCGCTGCTGACCGCGAGCAGCCAAGGTCCGCAGACCCTGTTTAGCCCGTTC
AACCAGTGGCCGGATTACGTTCTGCCGCTGGCGATCACCGTGCCGATCGTGGTTATTGTTCTGAGCGTGACCCTGGGTCTGGCGATCGGCATTCCGATGCACAAAAACAAGCAAGCGCTGA
AAGCGGGTTTTGCGCTGAGCAACCAGAAAGTTGACGTGCTGACCAAGGCGGTTGGTAGCGTGTTCAAGGAGATCATTAACCGTACCGGCATTAGCCAAGCGCCGAAACGTCTGAAGCAG
ACCAGCGCGGCGAAACCGGGTGCGCCGCGTCCGCCGGTTCCGCCGAAACCGGGCGCGCCGAAGCCGCCGGTGCAGCCGCCGAAAAAGCCGGCGTAA

Supplementary Table 4. Synthetic DNA for P40/P90

ATGAAGAGCAAACTGAAGCTGAAACGTTATCTGCTGTTCCTGCCGCTGCTGCCGCTGGGTACCCTGAGCCTGGCGAACACCTATCTGCTGCAAGATCACAACACCCTGACCCCGTACACCC

CGTTTACCACCCCGCTGAACGGTGGCCTGGATGTGGTTCGTGCGGCGCACCTGCACCCGAGCTATGAGCTGGTTGACTGGAAACGTGTTGGTGATACCAAACTGGTGGCGCTGGTTCGTAG

CGCGCTGGTGCGTGTTAAGTTCCAGGACACCACCAGCAGCGATCAAAGCAACACCAACCAGAACGCGCTGAGCTTTGACACCCAAGAAAGCCAGAAAGCGCTGAACGGTAGCCAAAGCG

GCAGCAGCGACACCAGCGGTAGCAACAGCCAGGATTTCGCGAGCTATGTGCTGATTTTTAAAGCGGCGCCGCGTGCGACCTGGGTTTTCGAGCGTAAGATCAAACTGGCGCTGCCGTACG

TGAAACAAGAAAGCCAGGGTAGCGGCGATCAGGGCAGCAACGGTAAAGGCAGCCTGTATAAGACCCTGCAAGACCTGCTGGTTGAGCAACCGGTTACCCCGTACACCCCGAACGCGGGT

CTGGCGCGTGTGAACGGTGTTGCGCAAGATACCGTTCACTTCGGTAGCGGCCAGGAAAGCAGCTGGAACAGCCAACGTAGCCAGAAGGGTCTGAAAAACAACCCGGGCCCGAAAGCGGT

TACCGGTTTTAAGCTGGACAAAGGCCGTGCGTATCGTAAGCTGAACGAGAGCTGGCCGGTGTACGAACCGCTGGACAGCACCAAAGAGGGTAAAGGCAAAGATGAAAGCAGCTGGAAG

AACAGCGAGAAAACCACCGCGGAAAACGACGCGCCGCTGGTTGGTATGGTTGGTAGCGGTGCGGCGGGTAGCGCGAGCAGCCTGCAGGGTAACGGCAGCAACAGCAGCGGTCTGAAAA

GCCTGCTGCGTAGCGCGCCGGTGAGCGTTCCGCCGAGCAGCACCAGCAACCAGACCCTGAGCCTGAGCAACCCGGCGCCGGTGGGCCCGCAAGCGGTGGTTAGCCAACCGGCGGGTGGC

GCGACCGCGGCGGTGAGCGTTAACCGTACCGCGAGCGATACCGCGACCTTCAGCAAGTACCTGAACACCGCGCAAGCGCTGCACCAGATGGGTGTGATCGTTCCGGGCCTGGAGAAATG

GGGTGGCAACAACGGTACCGGCGTGGTTGCGAGCCGTCAAGACGCGACCAGCACCAACCTGCCGCATGCGGCGGGTGCGAGCCAGACCGGTCTGGGTACCGGTAGCCCGCGTGAGCCGG

CGCTGACCGCGACCAGCCAACGTGCGGTTACCGTTGTTGCGGGTCCGCTGCGTGCGGGTAACAGCAGCGAAACCGACGCGCTGCCGAACGTGATTACCCAGCTGTATCACACCAGCACCG

CGCAACTGGCGTACCTGAACGGTCAGATCGTGGTTATGGGCAGCGACCGTGTTCCGAGCCTGTGGTACTGGGTGGTTGGCGAGGATCAAGAAAGCGGCAAAGCGACCTGGTGGGCGAAG

ACCGAGCTGAACTGGGGTACCGATAAGCAAAAACAGTTCGTGGAAAACCAGCTGGGCTTTAAGGACGATAGCAACAGCGACAGCAAAAACAGCAACCTGAAGGCGCAAGGTCTGACCC

AGCCGGCGTATCTGATTGCGGGCCTGGACGTGGTTGCGGATCACCTGGTGTTCGCGGCGTTTAAAGCGGGTGCGGTTGGCTATGACATGACCACCGATAGCAGCGCGAGCACCTACAACC

AAGCGCTGGCGTGGAGCACCACCGCGGGTCTGGACAGCGATGGTGGCTACAAGGCGCTGGTTGAAAACACCGCGGGTCTGAACGGCCCGATCAACGGTCTGTTCACCCTGCTGGATACCT

TTGCGTATGTGACCCCGGTTAGCGGCATGAAAGGTGGCAGCCAAAACAACGAGGAAGTGCAGACCACCTACCCGGTTAAGAGCGACCAGAAAGCGACCGCGAAGATTGCGAGCCTGATC

AACGCGAGCCCGCTGAACAGCTATGGTGACGATGGCGTGACCGTTTTCGATGCGCTGGGTCTGAACTTCAACTTTAAGCTGAACGAGGAACGTCTGCCGAGCCGTACCGACCAACTGCTG

GTGTACGGCATTGTTAACGAGAGCGAACTGAAAAGCGCGCGTGAGAACGCGCAGAGCACCAGCGACGATAACAGCAACACCAAGGTTAAATGGACCAACACCGCGAGCCACTACCTGCC

GGTGCCGTACTATTACAGCGCGAACTTTCCGGAGGCGGGTAACCGTCGTCGTGCGGAACAACGTAACGGCGTGAAGATCAGCACCCTGGAAAGCCAGGCGACCGATGGTTTCGCGAACA

GCCTGCTGAACTTTGGCACCGGTCTGAAGGCGGGTGTGGACCCGGCGCCGGTTGCGCGTGGCCACAAGCCGAACTATAGCGCGGTGCTGCTGGTTCGTGGTGGCGTGGTTCGTCTGAACTT

TAACCCGGACACCGATAAACTGCTGGACAGCACCGATAAGAACAGCGAGCCGATTAGCTTCAGCTACACCCCGTTTGGTAGCGCGGAAAGCGCGGTGGACCTGACCACCCTGAAAGATG

TTACCTATATCGCGGAGAGCGGCCTGTGGTTCTACACCTTTGACAACGGTGAAAAACCGACCTATGATGGCAAGCAGCAACAGGTTAAGAACCGTAAAGGTTACGCGGTGATTACCGTTA

GCCGTACCGGCATCGAGTTCAACGAAGATGCGAACACCACCACCCTGAGCCAGGCGCCGGCGGCGCTGGCGGTGCAAAACGGTATTGCGAGCAGCCAGGACGATCTGACCGGCATCCTG

CCGCTGAGCGACGAGTTCAGCGCGGTGATTACCAAGGACCAAACCTGGACCGGTAAAGTTGATATCTATAAGAACACCAACGGCCTGTTTGAGAAAGACGATCAGCTGAGCGAAAACGT

GAAGCGTCGTGACAACGGTCTGGTTCCGATCTACAACGAAGGTATCGTGGACATTTGGGGCCGTGTTGATTTTGCGGCGAACAGCGTTCTGCAGGCGCGTAACCTGACCGACAAGACCGT

GGATGAGGTTATTAACAACCCGGACATCCTGCAAAGCTTCTTTAAGTTCACCCCGGCGTTTGATAACCAGCGTGCGATGCTGGTTGGTGAAAAAACCAGCGACACCACCCTGACCGTTAA

GCCGAAAATTGAGTATCTGGACGGTAACTTCTACGGCGAAGATAGCAAAATCGCGGGTATTCCGCTGAACATTGATTTTCCGAGCCGTATCTTTGCGGGTTTTGCGGCGCTGCCGAGCTGG

GTGATCCCGGTGAGCGTTGGTAGCAGCGTTGGCATCCTGCTGATTCTGCTGATCCTGGGCCTGGGTATCGGCATTCCGATGTATAAGGTTCGTAAACTGCAAGACAGCAGCTTCGTGGATG

TTTTTAAGAAAGTGGACACCCTGACCACCGCGGTGGGTAGCGTTTACAAGAAAATCATTACCCAGACCAGCGTGATCAAGAAAGCGCCGAGCGCGCTGAAAGCGGCGAACAACGCGGCG

CCGAAAGCGCCGGTTAAACCGGCGGCGCCGACCGCGCCGCGTCCGCCGGTGCAGCCGCCGAAGAAAGCGTAA,

Supplementary Table 5. Primers used in this study

Primers	Sequence
**P1F**	AGGAGATATACCATGACCGTGGTTGGTCACTTTACC
**P1R**	GTGATGGTGATGTTTATCCGGCCACTGGTTGAACGG
**P40P90F**	AGGAGATATACCATGAGCCTGGCGAACACCTATCTGCTG
**P40P90GR**	GTGATGGTGATGTTTAACCAGACCGTTGTCACGACGC
**P40P90R**	GTGATGTGTATGTTTGCTCGGCACGCGCCGCAAAACC
**P40P90xINS1F**	GGTATGGTTGGTAGCGCGAGCGATACCGCGACC
**P40P90xINS1R**	CGCGGTATCGCTCGCGCTACCAACCATACCAAC
**P40P90xINS1&2F**	GTTCCGGGCCTGGAGAGAGCGAAACCGACGCG
**P40P90xINS1&2R**	GTCGGTTTCGCTGCTCTCCAGGCCCGGAACGATC
**P40P90RA445SSF**	ACCGCGACCAGCCAAAGTTCGGTTACCGTTGTTGCG
**P40P90RA445SSR**	CGCAACAACGGTAACCGAACTTTGGCTGGTCGCGGT
**P40P90RA455SSF**	GTTGCGGGTCCGCTGAGTTCGGGTAACAGCAGCGAAACC
**P40P90RA455SSR**	GGTTTCGCTGCTGTTACCCGAACTCAGCGGACCCGCAAC
**C-Domain K1376F**	AGGAGATATACCATGAAAATGAACGATGACGTTG
**C-Domain A1400F**	AGGAGATATACCATGGCGGATACCGGTCCGCAG

Supplementary References

1. Robert, X. & Gouet, P. Deciphering key features in protein structures with the new ENDscript server. *Nucleic acids research* **42**, W320-W324 (2014).
2. Burke, D.F., Deane, C.M. & Blundell, T.L. Browsing the SLoop database of structurally classified loops connecting elements of protein secondary structure. *Bioinformatics* **16**, 513-9 (2000).
3. Layh-Schmitt, G. & Herrmann, R. Localization and biochemical characterization of the ORF6 gene product of the Mycoplasma pneumoniae P1 operon. *Infect Immun* **60**, 2906-13 (1992).
4. Dallo, S.F., Su, C.J., Horton, J.R. & Baseman, J.B. Identification of P1 gene domain containing epitope(s) mediating Mycoplasma pneumoniae cytoadherence. *J Exp Med* **167**, 718-23 (1988).
5. Gerstenecker, B. & Jacobs, E. Topological mapping of the P1-adhesin of Mycoplasma pneumoniae with adherence-inhibiting monoclonal antibodies. *J Gen Microbiol* **136**, 471-6 (1990).
6. Jacobs, E., Pilatschek, A., Gerstenecker, B., Oberle, K. & Bredt, W. Immunodominant epitopes of the adhesin of Mycoplasma pneumoniae. *J Clin Microbiol* **28**, 1194-7 (1990).
