# Supplementary material for: Immunodominant proteins P1 and P40/P90 from human pathogen Mycoplasma pneumoniae
Source: Nat Commun. 2020 Oct 14;11:5188. doi: 10.1038/s41467-020-18777-y (PMC7560827; doi:10.1038/s41467-020-18777-y)
Supplement: Supplementary file 3 — Reporting Summary [file 41467_2020_18777_MOESM3_ESM.pdf]

nature research

Corresponding author(s): David Aparicio

Last updated by author(s): Aug 27, 2020

Reporting Summary

Nature Research wishes to improve the reproducibility of the work that we publish. This form provides structure for consistency and transparency in reporting. For further information on Nature Research policies, see our Editorial Policies and the Editorial Policy Checklist.

Statistics

For all statistical analyses, confirm that the following items are present in the figure legend, table legend, main text, or Methods section.

n/a	Confirmed	
☐	☒	The exact sample size (*n*) for each experimental group/condition, given as a discrete number and unit of measurement
☐	☒	A statement on whether measurements were taken from distinct samples or whether the same sample was measured repeatedly
☐	☒	The statistical test(s) used AND whether they are one- or two-sided<br>*Only common tests should be described solely by name; describe more complex techniques in the Methods section.*
☒	☐	A description of all covariates tested
☐	☒	A description of any assumptions or corrections, such as tests of normality and adjustment for multiple comparisons
☐	☒	A full description of the statistical parameters including central tendency (e.g. means) or other basic estimates (e.g. regression coefficient) AND variation (e.g. standard deviation) or associated estimates of uncertainty (e.g. confidence intervals)
☐	☒	For null hypothesis testing, the test statistic (e.g. *F*, *t*, *r*) with confidence intervals, effect sizes, degrees of freedom and *P* value noted<br>*Give P values as exact values whenever suitable.*
☒	☐	For Bayesian analysis, information on the choice of priors and Markov chain Monte Carlo settings
☒	☐	For hierarchical and complex designs, identification of the appropriate level for tests and full reporting of outcomes
☒	☐	Estimates of effect sizes (e.g. Cohen's *d*, Pearson's *r*), indicating how they were calculated

Our web collection on statistics for biologists contains articles on many of the points above.

Software and code

Policy information about availability of computer code

Data collection	The code is referenced. Biacore Control Software 3.0, Wyatt technology ASTRA-6, Wyatt technology Dynamics 7.0, 3918 Triturus, MXCuBe3
Data analysis	The code is referenced. Freeware (Coot 0.8, Xia2 0.3.6, Pymol 1.8.6, SerialEM, Relion 3.0 beta, Chimera 1.14, DMMulti 0.9, Biacore Evaluation Version 3.0, Wyatt technology Calypso 1.1, http://ekhidna2.biocenter.helsinki.fi/dali/)

For manuscripts utilizing custom algorithms or software that are central to the research but not yet described in published literature, software must be made available to editors and reviewers. We strongly encourage code deposition in a community repository (e.g. GitHub). See the Nature Research guidelines for submitting code & software for further information.

Data

Policy information about availability of data

All manuscripts must include a data availability statement. This statement should provide the following information, where applicable:

- Accession codes, unique identifiers, or web links for publicly available datasets
- A list of figures that have associated raw data
- A description of any restrictions on data availability

Atomic coordinates and structure factors for the reported crystal structures from P1, P40/P90, P40/P90-3SL and P40/P90-6SL have been deposited into the Protein Data Bank under accession codes 6RC9, 6RJ1, 6TLZ and 6TM0, respectively. Cryo-electron microscopy densities were deposited in the EM Databank under the accession code EMD-30233. The atomic coordinates of the Cryo-electron microscopy structure have been deposited into the Protein Data Bank under accesion code 7BWM.

Field-specific reporting

Please select the one below that is the best fit for your research. If you are not sure, read the appropriate sections before making your selection.

- [x] Life sciences
- [ ] Behavioural & social sciences
- [ ] Ecological, evolutionary & environmental sciences

For a reference copy of the document with all sections, see nature.com/documents/nr-reporting-summary-flat.pdf

Life sciences study design

All studies must disclose on these points even when the disclosure is negative.

Sample size	ELISA: Sample size was determined to ensure that the assay will yield results with the desired precision.
Data exclusions	ELISA: No data were excluded from the analysis
Replication	ELISA: Samples were tested in duplicates
Randomization	ELISA: Random allocation of samples
Blinding	ELISA: Investigators were not blinded

Reporting for specific materials, systems and methods

We require information from authors about some types of materials, experimental systems and methods used in many studies. Here, indicate whether each material, system or method listed is relevant to your study. If you are not sure if a list item applies to your research, read the appropriate section before selecting a response.

Materials & experimental systems

n/a	Involved in the study	
[ ]	[x]	Antibodies
[x]	[ ]	Eukaryotic cell lines
[x]	[ ]	Palaeontology and archaeology
[x]	[ ]	Animals and other organisms
[x]	[ ]	Human research participants
[ ]	[x]	Clinical data
[x]	[ ]	Dual use research of concern

Methods

n/a	Involved in the study	
[x]	[ ]	ChIP-seq
[x]	[ ]	Flow cytometry
[x]	[ ]	MRI-based neuroimaging

Antibodies

Antibodies used	Goat anti-Human IgG Fc Secondary Ab, HRP (ThermoFisher Scientific) Catalog H10007, Lot number 2020763A, RRID AB_2536544, Anti-adhesin P1 from Mycoplasma pneumoniae; not commercially available
Validation	Western Blot validation

Clinical data

Policy information about clinical studies

All manuscripts should comply with the ICMJEguidelines for publication of clinical research and a completedCONSORT checklist must be included with all submissions.

Clinical trial registration	The study was not registered as it doesn't correspond to a clinical trial
Study protocol	The study protocol is not available online but it can be requested to the authors or the ethics committee from the participating hospitals
Data collection	No clinical and personal data were collected apart from the clinical diagnosis and the IgG and IgM anti-M.pneumoniae values
Outcomes	To validate the presence in serum of patients infected with M. pneumoniae of high antibody titers against candidate immunodominate epitopes present of adhesin proteins by ELISA
